# Predicting who takes music lessons: parent and child characteristics

**DOI:** 10.3389/fpsyg.2015.00282

**Published:** 2015-03-24

**Authors:** Kathleen A. Corrigall, E. Glenn Schellenberg

**Affiliations:** ^1^Department of Psychology, MacEwan UniversityEdmonton, AB, Canada; ^2^Department of Psychology, University of Toronto MississaugaMississauga, ON, Canada

**Keywords:** music training, music lessons, cognition, personality, individual differences, gene-environment interaction

## Abstract

Studies on associations between music training and cognitive abilities typically focus on the possible benefits of music lessons. Recent research suggests, however, that many of these associations stem from niche-picking tendencies, which lead certain individuals to be more likely than others to take music lessons, especially for long durations. Because the initial decision to take music lessons is made primarily by a child's parents, at least at younger ages, we asked whether individual differences in parents' personality predict young children's duration of training. Children between 7 and 9 years of age (*N* = 170) with varying amounts of music training completed a measure of IQ. Their parents provided demographic information as well as ratings of their own and their child's Big Five personality dimensions. Children's personality traits predicted duration of music training even when demographic variables and intelligence were held constant, replicating findings reported previously with 10- to 12-year-olds and 17-year-olds. A novel finding was that parents' openness-to-experience predicted children's duration of training, even when characteristics that pertained to children (demographic variables, intelligence, and personality) were controlled statistically. Our findings are indicative of passive and active gene-environment correlations, whereby genetic predispositions influence the likelihood that a child will have certain experiences, such as music training.

## Introduction

What kinds of parents choose to enroll their children in music lessons, and how do children who take music lessons differ from their peers? Typically, parents take primary responsibility for the decision to enroll young children in an extracurricular activity. As children grow up and become more independent, they gain more control over choosing their activities—deciding whether to continue pursuing their current extracurricular activities or to pursue new interests. To date, most research has focused on associations between music training and non-musical abilities in the interest of exploring the potential benefits of music training. The present investigation, by contrast, asked whether pre-existing individual differences determine who takes music lessons. In other words, we asked how genes interact with the environment.

Until recently, few studies examined factors that influence the likelihood of taking music lessons or developing musical expertise, and most of the available research was conducted with adults rather than children. Adults who achieve high levels of musical expertise differ from less accomplished musicians in terms of greater levels of passion for music, enhanced music aptitude, and more practice (e.g., Ericsson et al., 1993; Ruthsatz et al., 2008; Bonneville-Roussy et al., 2011; Macnamara et al., 2014). In fact, practice is the best predictor that children and adolescents will continue playing music into adulthood (Theorell et al., 2015). Practice is also associated with the propensity to experience musical flow, which is defined as being completely absorbed and focused on an activity that one enjoys (Butkovic et al., 2015). Importantly, most of these individual differences appear to be genetically influenced, including music aptitude (Drayna et al., 2001; Ukkola et al., 2009; Park et al., 2012; Ukkola-Vuoti et al., 2013; Mosing et al., 2014; Tan et al., 2014), musical achievement (Hambrick and Tucker-Dobb, 2015), musical flow (Butkovic et al., 2015), and even practice (Mosing et al., 2014; Butkovic et al., 2015; Hambrick and Tucker-Dobb, 2015). These findings suggest those who are most involved with and skilled at music may be naturally inclined to seek out environments that match their predispositions.

Demographic variables also play a role. Compared to musically untrained individuals, those who take music lessons tend to come from families with higher socioeconomic status (SES; Sergeant and Thatcher, 1974; Schellenberg, 2006, 2011a,b; Corrigall et al., 2013; Müllensiefen et al., 2014), and to have greater involvement in non-musical extracurricular activities (Schellenberg, 2006, 2011a; Corrigall et al., 2013). In short, children who take music lessons tend to have parents who can afford to pay for the lessons and for other extracurricular activities. High-SES parents may also place greater value on learning opportunities outside of regular school, and therefore support children's continued participation in extracurricular activities.

Music training, expertise, and practice are all associated with openness-to-experience, a Big Five trait that involves an interest in the arts, intellectual curiosity, and a tendency to try new activities and learn various skills, including how to play a musical instrument (Dyce and O'Connor, 1994; Gillespie and Myors, 2000; Corrigall et al., 2013; Hille and Schupp, 2014; Müllensiefen et al., 2014; Butkovic et al., 2015). Because personality traits are also genetically influenced (Matthews et al., 2003; Bouchard, 2004), these findings are consistent with the idea that certain individuals are more likely to take music lessons in the first place and to continue their involvement in music, such that these experiences allow them to become highly skilled. More generally, individuals who are high in openness-to-experience seek out experiences that help them learn new skills, a gene-environment interaction that ultimately enhances cognitive functioning and achievement (Dollinger and Orf, 1991; McCrae, 1993; Harris, 2004; Moutafi et al., 2006; John et al., 2008).

The prevailing view, however, is that music lessons enhance non-musical abilities such as speech perception (Strait and Kraus, 2011), working memory (George and Coch, 2011), and executive functions (Bialystok and DePape, 2009; Moradzadeh et al., 2014), with little to no consideration of pre-existing individual differences that influence who takes music lessons in the first place. This view has filtered down to the general public. For example, when parents are asked why it is important for their children to have music training, they often mention cognitive benefits such as improving intelligence and academic performance (Dai and Schader, 2001; Mehr, 2014). Indeed, it is well-documented that music training is associated positively with many cognitive skills (see Costa-Giomi, 2012; Schellenberg and Weiss, 2013 for reviews). Because the vast majority of these findings came from studies with correlational or quasi-experimental designs, the view that music training is causing the associations represents a logical mistake of inferring causation from correlation, while ignoring the role of pre-existing differences in cognitive abilities, personality, music aptitude, and demographic background (Schellenberg, 2015).

Some researchers (e.g., Ho et al., 2003; Forgeard et al., 2008; George and Coch, 2011; Strait and Kraus, 2011) argue that associations between *duration* of music training and non-musical abilities constitute evidence for a dose-dependent benefit—the longer the training, the greater the improvement, even though pre-existing differences (re: cognition, personality, demographics) undoubtedly play a role in determining who takes music lessons for years on end (Corrigall et al., 2013). Correlations between age of onset of music training and task performance (or brain structure or function) are similarly interpreted as evidence for greater plasticity at younger ages (e.g., Habib and Besson, 2009; Penhune, 2011), even though children who begin music lessons at young ages are likely to differ systematically from other children. Finally, in longitudinal quasi-experiments, lack of evidence for an association at pre-test (Norton et al., 2005) does not guarantee that associations at post-test (Hyde et al., 2009) are solely the consequence of learning and the environment. Early in development, genetically determined behaviors and characteristics may not be evident phenotypically.

It is well-documented that the influence of genes on the environment changes as children develop (Plomin et al., 1977; Scarr and McCartney, 1983; Plomin, 2014). Such influences are initially *passive*, when the parents' genotype (related to the child's genotype) determines the environment in which a child is raised. As the child develops, the genetic influence becomes *evocative*: children's predispositions influence how others respond to them. As children age further, they start to play an *active* role, seeking out environments that match who they are, a process called “niche-picking.”

Corrigall et al. (2013) recruited a large sample of 10- to 12-year-olds and reported that duration of music training was associated positively with SES, intelligence, and participation in non-musical extracurricular activities, as well as with two personality traits: conscientiousness and openness-to-experience. Associations between music training and conscientiousness, and between music training and openness, were also evident in study of 17-year-olds (Hille and Schupp, 2014). These findings are consistent with an active gene-environment correlation: Bright, curious, and motivated individuals seek out intellectually enriching activities—including but not limited to music lessons—that are likely to make them even more bright, curious, and motivated. Because *passive* genetic effects play a larger role at younger ages, one would expect that parents' characteristics would more strongly influence whether younger children take music lessons.

In the present investigation, we tested this prediction in a group of 7- to 9-year-olds, who are at the age when traditional music lessons (i.e., private instrumental training such as piano lessons) typically begin. The children were administered a standardized measure of intelligence. Their parents provided demographic information as well as ratings of their child's and their own personalities. Demographic, cognitive, and personality variables were used in regression analyses to predict duration of music training. We expected to replicate findings that children's own characteristics (such as their cognitive abilities and personalities) predict the environments they experience, in this case music training (Corrigall et al., 2013; Degé et al., 2014; Hille and Schupp, 2014). Because our sample comprised children younger than those tested previously, we also expected parents' personalities to predict their children's duration of music training.

## Method

The study protocol was approved by the Research Ethics Board at the University of Toronto.

### Participants

We recruited 170 7- to 9-year-olds (88 girls, 82 boys, mean age 8.6 years, *SD* = 0.8 years) from the local community. Children received a gift certificate as appreciation for participating. Descriptive statistics are provided in Table 1.

**Table 1:** **Descriptive statistics for outcome and predictor variables**.

**Variable**	***M***	***SD***
Cumulative duration of music training in months	13.3	17.7
Age in years	8.6	0.8
Family income	5.6	2.1
Parents' education	5.5	1.6
Cumulative duration of non-musical activities in months	46.8	39.4
IQ	111.1	12.9
Average grade	8.7	1.2
Openness (C)	5.3	0.9
Conscientiousness (C)	4.6	0.8
Extraversion (C)	5.0	0.7
Agreeableness (C)	4.9	0.8
Neuroticism (C)	3.6	0.8
Openness (P)	3.5	0.6
Conscientiousness (P)	4.1	0.6
Extraversion (P)	3.6	0.8
Agreeableness (P)	4.1	0.5
Neuroticism (P)	2.6	0.7

*Family income was measured on a 9-point scale in increments of $25,000 (1 = less than $25,000; 2 = $25,000–$49,999; 3 = $50,000–$74,999; and so on). Parents' education was measured on an 8-point scale (1 = did not finish high school, 8 = graduate degree). Average grade was measured on a 12-point scale (where the highest grade = 12 and a failing grade = 0). Child personality traits were measured on 7-point scales and parent personality traits were measured on 5-point-scales in which higher scores indicate stronger trait tendencies. C, child; P, parent*.

### Outcome variable

The outcome variable was cumulative months of extracurricular music lessons (i.e., individual or group lessons outside of the regular school curriculum). Duration of training was summed for children who took both individual and group lessons, or who took lessons on two different instruments at the same time (e.g., a child who took both piano and violin lessons for 9 months simultaneously was considered to have 18 months of music training). For those with some training (*n* = 98), duration ranged from 0.5 to 88 months (*M* = 23.0 months; *SD* = 17.8 months), and 65% were still taking lessons at the time of the study (*n* = 64).

### Predictor variables

Annual family income was measured on a 9-point scale (1 ≤ $25,000, 9 ≥ $200,000; Canadian dollars, data missing for four children). The modal income was 4 ($75,000–$100,000); the median was 5 ($100,000–$125,000). Parents' education was measured on an 8-point scale (1 = did not finish high school, 8 = graduate degree). For both mothers and fathers, the modal and median response was 6 (university graduate), and responses were averaged across parents for subsequent analyses. Because music training also tends to be associated with involvement in other extracurricular activities (Schellenberg, 2006, 2011a; Corrigall et al., 2013), we collected information about participation in activities such as sports and arts other than music. Duration of involvement was summed as for music training. On average, children had 46.8 cumulative months of involvement in non-musical activities (*SD* = 39.4 months).

Children were administered the two-subtest version of the Wechsler Abbreviated Scale of Intelligence (WASI; Wechsler, 1999). As in previous research with middle-class Canadian children (Schellenberg, 2006, 2011a; Corrigall et al., 2013), the average IQ (*M* = 111.1, *SD* = 12.9) was substantially higher than American norms, *t*_(169)_ = 11.21, *p* < 0.001. Parents of 92% of the sample (157 children) provided photocopies of school report cards. In the province of Ontario, report cards for publicly funded schools have a standardized format in which grades are reported on the same scale. Children's grades were converted to a 12-point numerical scale (where the highest grade = 12 and a failing grade = 0) and averaged across school subjects (*M* = 8.69, *SD* = 1.18).

To measure children's personality, a parent completed the short version of the Inventory of Children's Individual Differences (ICID-S; Deal et al., 2007). Parents also provided self-reports of their own personality using the Big Five Inventory (BFI; John et al., 1991). Although parents' personality scores were significantly correlated with the scores they provided for their children (with the exception of conscientiousness), the weak to moderate correlations (*r*s ranged between 0.084 and 0.325, see Table 2) confirmed that parents made a distinction between their personalities and those of their children.

**Table 2:** **Correlations among predictor variables**.

Predictor variables		**2**	**3**	**4**	**5**	**6**	**7**	**8**	**9**	**10**	**11**	**12**	**13**	**14**	**15**	**16**	**17**
1. Age	−	0.140	0.071	0.029	0.257^**^	0.201^**^	−0.044	−0.053	−0.201^**^	−0.025	−0.003	0.013	0.017	−0.024	−0.054	−0.022	0.004
2. Gender		−	0.044	0.017	0.080	−0.061	−0.207^**^	−0.138	−0.251^**^	0.014	−0.104	0.106	−0.090	−0.016	−0.137	−0.124	−0.077
3. Family income			−	0.413^**^	0.296^**^	0.172^*^	0.308^**^	0.076	0.128	0.038	0.119	−0.117	0.079	0.191^*^	−0.009	−0.091	−0.216^**^
4. Parents' education				−	0.218^**^	0.007	0.288^**^	−0.049	0.040	−0.104	0.119	−0.080	0.070	0.265^**^	0.045	−0.003	−0.054
5. Non-musical activities					−	0.123	0.092	0.111	−0.001	0.165^*^	0.178^*^	−0.008	0.101	0.161^*^	0.059	−0.001	0.032
6. IQ						−	0.367^**^	0.374^**^	0.282^**^	0.140	0.136	−0.147	0.065	0.088	0.011	0.051	−0.094
7. Average grade							−	0.281^**^	0.439^**^	0.034	0.193^*^	−0.338^**^	−0.040	0.059	0.079	0.123	0.031
8. Openness (C)								−	0.626^**^	0.564^**^	0.355^**^	−0.417^**^	0.221^**^	0.044	0.156^*^	0.188^*^	−0.122
9. Conscientiousness (C)									−	0.260^**^	0.506^**^	−0.574^**^	0.108	0.084	0.052	0.150	−0.116
10. Extraversion (C)										−	0.367^**^	−0.432^**^	0.124	0.215^**^	0.178^*^	0.256^**^	−0.183^*^
11. Agreeableness (C)											−	−0.608^**^	0.175^*^	0.255^**^	0.065	0.325^**^	−0.157^*^
12. Neuroticism (C)												−	−0.064	−0.221^**^	−0.055	−0.204^**^	0.234^**^
13. Openness (P)													−	0.082	0.127	0.171^*^	−0.187^*^
14. Conscientiousness (P)														−	0.194^*^	0.263^**^	−0.315^**^
15. Extraversion (P)															−	0.305^**^	−0.209^**^
16. Agreeableness (P)																−	−0.266^**^
17. Neuroticism (P)																	−

*C, child; P, parent*.

* p < 0.05;

** *p < 0.01*.

### Procedure

Children were administered the WASI by a trained research assistant. A parent completed a demographics questionnaire, the ICID-S as it pertained to the child, and the BFI as self-report.

## Results

Pairwise correlations among the predictor variables are provided in Table 2. The principal analyses examined associations between the outcome variable (duration of music training) and the predictor variables. Preliminary inspection of scatterplots indicated no violations of linearity. Pairwise correlations revealed that children who took music lessons for longer durations tended to be older, *r* = 0.20, *N* = 170, *p* = 0.008, to come from families with higher incomes, *r* = 0.23, *N* = 166, *p* = 0.003, to have parents with more education, *r* = 0.15, *N* = 170, *p* = 0.006, and to be more involved in non-musical activities, *r* = 0.19, *N* = 170, *p* = 0.013. They also tended to have higher IQs, *r* = 0.21, *N* = 170, *p* = 0.006, and to be more open-to-experience, *r* = 0.24, *N* = 170, *p* = 0.002, and agreeable, *r* = 0.25, *N* = 170, *p* = 0.001, but less neurotic, *r* = −0.15, *N* = 170, *p* = 0.045. Finally, children with more music training had parents who were more open-to-experience, *r* = 0.30, *N* = 170, *p* < 0.001. In absolute terms, this final association was the strongest we observed.

Identification of pairwise associations between duration of music training and several predictor variables motivated further analyses to determine which of these predictors had significant partial associations with duration of training. Hierarchical multiple regression was used with duration of music training as the outcome variable. Predictor variables were those that had simple associations with duration of music training (Table 3). Demographic variables (age, family income, parent's education, and duration of non-musical activities) and IQ were entered on the first step, which together accounted for 11.4% of the variance in duration of music training (adjusted *R*^2^ = 0.086). Partial correlations revealed that age, family income, and IQ were marginally significant predictors (*p*s < 0.10). Thus, the association between duration of music training and IQ was near-significant when demographic variables were held constant, although it was weaker than in previous correlational studies with older children (Schellenberg, 2006, 2011a; Corrigall et al., 2013) and in a quasi-experiment with same-age children (Schellenberg and Mankarious, 2012).

**Table 3:** **Results from hierarchical multiple regression with duration of music training as the outcome variable**.

**Predictor**	**Partial correlation (*pr*)**	***p*-value**
**Step 1: *R* = 0.337, adjusted *R*^2^ = 0.086, *F*_(5, 160_)** = 4.10, *p* = 0.002		
Age	0.13	0.093
Family income	0.14	0.083
Parents' education	0.06	0.457
Non-musical activities	0.08	0.323
IQ	0.15	0.063
**Step 2: *R* = 0.416, adjusted *R*^2^ = 0.131, *F*_(8, 157)_** = 4.11, *p* < 0.001		
Age	0.17	0.029
Family income	0.14	0.082
Parents' education	0.07	0.414
Non-musical activities	0.02	0.770
IQ	0.06	0.441
Child's openness-to-experience	0.14	0.084
Child's agreeableness	0.17	0.031
Child's neuroticism	0.05	0.498
**Step 3: *R* = 0.469, adjusted *R*^2^ = 0.175, *F*_(9, 156)_** = 4.90, *p* < 0.001		
Age	0.17	0.029
Family income	0.13	0.093
Parents' education	0.06	0.461
Non-musical activities	0.02	0.838
IQ	0.07	0.384
Child's openness-to-experience	0.10	0.228
Child's agreeableness	0.15	0.070
Child's neuroticism	0.03	0.702
Parent's openness-to-experience	0.24	0.003

We added children's personality variables (openness-to-experience, agreeableness, and neuroticism) on the second step to examine whether personality traits helped to explain duration of music training when demographic and cognitive variables were held constant. Children's personality accounted for an additional 5.9% of the variance in duration of music training, *F*_inc(3,157)_ = 3.76, *p* = 0.031, with the new model accounting for 17.3% of the variance in duration of music lessons (adjusted *R*^2^ = 0.131). Age and the child's agreeableness were significant predictors, whereas family income and the child's openness-to-experience made marginal contributions. As in previous research (Corrigall et al., 2013), accounting for personality variables in addition to demographic variables rendered the association between IQ and duration of music training non-significant.

On the third step, parents' personality (openness-to-experience) was added to the model to examine whether parent characteristics helped to explain how long their children took music lessons when variables that pertain to the child (demographics, cognitive ability, personality) were held constant. The addition of parents' openness-to-experience improved explanatory power by an additional 4.7%, *F*_inc(1,156)_ = 9.42, *p* = 0.003, such that the model now accounted for 22.0% of the variance in duration of training (adjusted *R*^2^ = 0.175). Age and parents' openness-to-experience made significant contributions, and family income and children's agreeableness made marginal contributions.

## Discussion

We examined whether duration of music training was associated with demographic, cognitive, and personality variables in a large sample of 7- to 9-year-old children. We hypothesized that at these young ages, parental characteristics would be associated with children's duration of training. As duration of training increased, so did SES, IQ, and involvement in non-musical extracurricular activities. Duration of training was also associated positively with children's openness-to-experience and agreeableness, negatively with children's neuroticism, and positively with the parents' openness-to-experience. The sheer number of association confirms that children who take music lessons differ from other children in many respects.

As in an earlier study (Corrigall et al., 2013), children's personality predicted duration of music training even after demographic and cognitive abilities were held constant, but the association between music training and intelligence disappeared when demographic variables and personality were statistically controlled. Because individual differences in personality are strongly heritable (Bouchard, 2004), the present findings considered jointly with the earlier results point to gene-environment interactions, in which the child's role becomes increasingly active over development (Scarr and McCartney, 1983). Early in development, the parents' openness-to-experience is the principle factor determining whether their children take music lessons, consistent with passive genetic influences. At this point, the children's agreeableness matters most, because they must agree to go along with their parents' decision. For older children who are more independent, their own level of openness-to-experience is the principal factor that determines whether they continue taking lessons. Throughout this process, music lessons are an environmental factor that is consistent with the child's predispositions, which are, in turn, reinforced and enhanced by the environment.

In the earlier study, an association between duration of music training and conscientiousness in 10- to 12-year-olds explained why musically trained children do better in school than one would predict based on IQ (Corrigall et al., 2013). Musically trained 10- to 12-year-olds are particularly hard-working, diligent, and self-disciplined, traits that facilitate performance in school. In the present sample of 7- to 9-year-olds, duration of music training was not associated with children's conscientiousness or with their average grade in school. One possibility is that conscientiousness influences children's tendency to *persist* at music lessons, but not whether they take lessons in the first place or begin lessons early in life. As such, children in our sample may have been too young for conscientiousness to exert much influence. Rather, duration of training at this age was associated with higher levels of agreeableness and lower levels of neuroticism. Because young children's participation in extracurricular activities is controlled primarily by their parents, these associations could reflect children's tendency to be cooperative and compliant (related to agreeableness), and “easy-going” rather than hostile, anxious, and impulsive (related to neuroticism). It is also possible that we found a different pattern of associations because children's personality traits were more strongly inter-correlated (see Table 2) than they were for the older children tested by Corrigall et al. (2013). In general, personality traits become more distinct as children develop (e.g., Caspi et al., 2005; Soto et al., 2008).

Why is openness-to-experience the best overall predictor of music training, whether it comes from the child or the parent? One obvious reason is that part of openness-to-experience is *aesthetic sensitivity* (i.e., liking and appreciating music, art, and dance). Openness is also associated with creativity (McCrae, 1987), intelligence (McCrae, 1993; Harris, 2004; Moutafi et al., 2006), academic achievement (Dollinger and Orf, 1991; John et al., 2008), and an interest in and enjoyment of intellectual activities (Furnham et al., 2008). It is associated with variation in two genes involved in dopaminergic function in the prefrontal cortex (DeYoung et al., 2011). The prefrontal dopamine system is known to be associated with working memory (e.g., Braver and Cohen, 2000; Robbins, 2005), learning about rewards (Schultz, 2006), and motivation to understand or know the answer to a question (Panksepp, 1998).

As noted, there is a genetic component to music-specific abilities (Tan et al., 2014), including the perception of tonality (Drayna et al., 2001) and music aptitude (Ukkola et al., 2009; Park et al., 2012; Ukkola-Vuoti et al., 2013). Although expert levels of performance have been claimed to be a consequence of deliberate practice (Ericsson et al., 1993), empirical evidence indicates that that role of practice accounts for only 21% of the variance in music performance (Macnamara et al., 2014). Moreover, music practice has a large genetic contribution (38%), as does musical accomplishment (26%), and the genetic component to accomplishment is largely independent of the genetic component to practice (Hambrick and Tucker-Dobb, 2015). Finally, each Big Five personality dimension has a heritability estimate of approximately 50%, whereas heritability estimates of IQ increase steadily during development, reaching a high of over 80% in adulthood (Bouchard, 2004). In short, music training is unlikely to have large and systematic non-musical benefits that are consistent across individuals regardless of their genetic make-up.

Research on music training should be considered within the cultural context in which it takes place—namely, Western cultures. Because learning to play music in the Western world typically involves relatively costly individual lessons taken outside of school, training on an expensive instrument (e.g., piano), and school-like, accomplishment-driven pedagogies (e.g., music conservatory exams and grades), pre-existing differences may exert a larger influence than they would in cultures in which music learning and performance are more integrated into daily life. In many non-Western cultures, music making is a culturally valued and frequent group activity in which all members of the group participate, such as in rituals or celebrations (Sloboda, 1988; Dunbar et al., 2012). In these contexts, variables such as SES, intelligence, and personality may play a much smaller role in influencing which individuals learn to play music.

Our findings highlight the need to consider predispositions when studying the potential benefits of environmental interventions such as music training. We do not deny that environmental effects play a role in any behavior, but a complete account requires consideration of genetics as well. In quasi-experimental and correlational studies, associations between music training and non-musical skills are likely to reflect gene-environment interactions rather than simple environmental effects (Schellenberg, 2015). In true experiments using random assignment to music lessons or a control group, inconsistent findings (Bilhartz et al., 1999; Costa-Giomi, 1999; Schellenberg, 2004; Moreno et al., 2009, 2011; Kaviani et al., 2014) may reflect the tendency for certain individuals—such as those who are high in openness-to-experience—to reap more benefit from stimulating environments. Future research on associations between music training and non-musical abilities in childhood should consider individual differences that pertain to parents (such as personality and cognitive ability) as well as those that pertain to children.

### Conflict of interest statement

The Guest Associate Editor Franziska Degé declares that, despite having collaborated with author E. Glenn Schellenberg, the review process was handled objectively and no conflict of interest exists. The authors declare that the research was conducted in the absence of any commercial or financial relationships that could be construed as a potential conflict of interest.
